# A multi-anatomical *in vivo* and *in vitro* ultrafast ultrasound dataset: 20,431 acquisitions with 105 plane waves using the GE 11L-D probe and a vantage system

**DOI:** 10.1016/j.dib.2026.113259

**Published:** 2026-09-15

**Authors:** Roser Viñals, Jean-Philippe Thiran

**Affiliations:** Signal Processing Laboratory 5, École Polytechnique Fédérale de Lausanne, 1015, Lausanne, Switzerland

**Keywords:** Beamforming, Coherent compounding, Deep learning, Image reconstruction, Medical imaging, Rf signals, Unfocused wavefronts

## Abstract

This article presents a comprehensive, multi-anatomical raw radio frequency (RF) ultrafast ultrasound dataset designed to facilitate advancements in beamforming, image reconstruction, and deep learning-based medical imaging. The data were acquired using a GE 11L-D linear array probe (192 elements, 7.3 MHz center frequency) interfaced with a Verasonics Vantage 256 system. The dataset comprises 20,431 total acquisitions, providing a diverse set of RF signals across varying tissue types. This includes 20,121 *in vivo* acquisitions collected from 16 healthy volunteers across six distinct anatomical regions—abdomen, carotids, breasts, back, upper limbs, and lower limbs—as well as 310 *in vitro* acquisitions from a CIRS 054GS phantom. Each acquisition consists of 105 plane waves (PWs) with a steering angle spacing of 0.32°, which enables coherent plane wave compounding (CPWC) for high-quality image reconstruction. All data collection was conducted under strict ethical safety guidelines approved by the Cantonal Commission on Ethics in Human Research (CER-VD, Switzerland). By providing a large-scale, heterogeneous dataset of raw signals, this dataset addresses the critical scarcity of open-access raw ultrasound data. It serves as a robust benchmark for evaluating traditional beamforming algorithms, validating novel image reconstruction techniques, and training data-intensive deep learning models. This resource is intended to foster reproducibility and accelerate research in medical ultrasound imaging.

Specifications Table

**Table:** 

Subject	Computer Sciences
Specific subject area	Ultrafast ultrasound imaging, signal processing, deep learning, and image reconstruction in medical imaging*.*
Type of data	Raw radio frequency (RF) signals, Metadata, Beamforming parameters, Sequence configuration.
Data collection	Acquisitions were performed using a GE 11L-D probe and a Verasonics Vantage 256 system. A total of 20,121 *in vivo* acquisitions were obtained from 16 healthy volunteers (6 anatomical regions) and 310 acquisitions of a CIRS 054GS phantom. Each acquisition consists of a sequence of 105 plane waves (PWs) with a 0.32° steering spacing*.*
Data source location	Signal Processing Laboratory 5 (LTS5), École Polytechnique Fédérale de Lausanne (EPFL), Lausanne, Switzerland
Data accessibility	Repository name: Zenodo Data identification number: 10.5281/zenodo.20054330 Direct URL to data: https://doi.org/10.5281/zenodo.20054330 Instructions for accessing these data: The Zenodo repository serves as the official landing page for the dataset metadata and documentation [1]. Due to the large volume of data, the raw RF files are hosted on dedicated EPFL servers to ensure high-speed access and efficient data management. The Zenodo record provides the permanent Digital Object Identifier (DOI), comprehensive metadata, acquisition parameters, and documentation, along with detailed instructions for accessing and downloading the raw RF data via direct links or S3-compatible interfaces.
Related research article	R. Viñals and J. -P. Thiran, ``Deep Learning-Based Inpainting for Sparse Arrays in Ultrafast Ultrasound Imaging,'' in IEEE Transactions on Computational Imaging, vol. 12, pp. 187–202, 2026, doi:10.1109/TCI.2025.3648531 [2].

## 1 Value of the Data

• Large-scale diversity: With over 20,000 acquisitions, this dataset provides the critical volume of data required to train and validate deep learning architectures in ultrasound imaging.
• The inclusion of six distinct anatomical regions (upper and lower limbs, back, breast, carotids, and abdomen) enables the development of generalized models that are robust to varying tissue textures.
• Raw RF data accessibility: While many public datasets are limited to post-processed B-mode or RF images, this repository provides raw RF signals. This enables the scientific community to develop and benchmark new algorithms for fundamental stages of the imaging chain, such as image reconstruction.
• Standardized benchmark for image and sparse reconstruction: The sequence of 105 PWs can be used to generate high-quality target images via coherent plane wave compounding (CPWC). Researchers use this data to develop novel reconstruction methods, deep learning methods, and can downsample the PW acquisitions to simulate sparse apertures, providing a standardized platform to compare the performance of different reconstruction techniques against a high-quality fully-sampled baseline [2].
• Foundation pre-training for transfer learning: While not restricted to a single clinical disease or specific pathology, this dataset serves as a general-purpose pre-training foundation for supervised and self-supervised learning approaches. Training deep networks on a diverse set of anatomical regions allows them to learn universal features and generalize effectively to entirely unseen anatomical structures [3]. Furthermore, training on broad *in vivo* data captures real-world acoustic phenomena (*e.g.*, phase aberration, multiple-scattering, attenuation) that physics-based simulations often fail to model accurately, yielding superior performance than training on simulated data [4]. Models pre-trained on this resource can subsequently be fine-tuned using small, task-specific datasets.
• Integrated phantom acquisitions: By including acquisitions from a CIRS 054GS multi-purpose phantom alongside the *in vivo* data, this dataset allows researchers to validate algorithm performance (*e.g.*, point spread function, axial and lateral resolution, and contrast) in a controlled medium.
• Reproducibility and longitudinal studies: The volunteer IDs are synchronized with a previous work and dataset published in [2], facilitating longitudinal algorithm development and cross-study comparisons. Furthermore, the provision of the original Verasonics sequence file and raw metadata ensures that the experimental setup can be exactly replicated or extended by other laboratories.
• Public cross-hardware benchmarking: Paired with the GE 9L-D dataset in [2], this resource enables independent, reproducible evaluations of domain adaptation and cross-probe transfer learning across different linear ultrasound transducers.

## 2 Background

The development of this dataset was motivated by the need for large-scale, raw ultrasound data to train and benchmark deep learning models for signal reconstruction. By acquiring 105 steered PWs across six anatomical regions, this dataset provides a statistically diverse range of acquisitions essential for robust model evaluation.

This data article adds significant value to the related research published in [2]. In the original study, a similar dataset acquired with a GE 9L-D probe was used for training, while only a small subset of the current GE 11L-D dataset was used to test model generalization. With this article, we release a complete, large-scale dataset of over 20,000 acquisitions acquired with the GE 11L-D transducer. This volume is significantly larger than the subset previously used in [2], providing sufficient diversity to train deep learning models from scratch, explore alternative sparse array strategies, image reconstruction methods, and image improvement models that extend beyond the scope of the original generalization study. In addition, by releasing this GE 11L-D dataset [1] alongside our previously published GE 9L-D dataset [2], we provide a complete multi-hardware benchmark that enables researchers to independently explore cross-transducer transfer learning and domain adaptation.

## 3 Data Description

The dataset is organized into four primary components: metadata and acquisition settings, a GPU-accelerated PyTorch beamforming script, *in vitro* data, and *in vivo* acquisitions. All files are hosted on an S3-compatible repository and *in vivo* and *in vitro* acquisitions are indexed via a master CSV file. The directory structure and file contents are described below.

### 3.1 Global metadata and acquisition settings

• **README.md:** Provides a high-level overview, download instructions, and sample code for data loading.
• **dataset.csv**: A master index in comma-separated value format. Each row contains the unique acquisition ID (matching the filename), the volunteer identifier (000 in case of *in vitro* acquisitions), and the region label, as detailed in Table 1.

**Table 1:** Master metadata dictionary for dataset.csv.

Field Name	Data Type	Description	Allowed / Example Values
acquisition_id	String	Unique identifier for each raw RF acquisition file in the dataset, formatted with a 5-digit zero-padded index.	acquisition_00001 to acquisition_20,431
volunteer_id	Integer	Anonymized numerical identifier assigned to human participants or set to 0 for phantom acquisitions.	0 (*In vitro*) 3–19 (*In vivo*)
body_region	String	Target anatomical location or phantom setting for the ultrasound acquisition.	*In vivo:* abdomen, arm, back, breast, carotid, leg *In vitro*: full_speckle, inclusions
• **beamform.py:** A GPU-accelerated PyTorch script performing delay-and-sum (DAS) beamforming, envelope extraction, and log compression to reconstruct B-mode images from raw acquisition files.
• **settings/**: A ZIP folder containing the hardware and sequence configuration files necessary for beamforming and signal processing:
  ◦ **beamforming_settings.yaml**: Contains the physical parameters of the GE 11L-D probe (pitch, element count, etc.) and the Verasonics Vantage 256 system settings in SI units.
  ◦ **steering_angles.npy**: A NumPy array containing the 105 steering angles (in radians) used in the PW sequence.
  ◦ **time_axis.npy / time_axis_per_angle.npy**: NumPy arrays defining the temporal sampling vectors [s] for the RF signals.
  ◦ **sequence_verasonics_ge11ld_105pws.mat**: The original MATLAB-based Verasonics sequence code.

### 3.2 Data folders

• **in_vitro**: Contains 310 acquisitions of the CIRS 054GS multi-tissue phantom, compressed in a ZIP file. This ZIP file contains two subfolders to facilitate different stages of image reconstruction and evaluation:
  ◦ full_speckle/: Contains acquisitions of uniform phantom regions showing only fully developed speckle. These datasets are intended for the derivation of normalization matrices [2,3].
  ◦ inclusions/: Contains acquisitions from the CIRS 054GS phantom featuring point targets and cyst-like inclusions. These files are specifically designed for evaluating standard imaging metrics, such as axial and lateral resolution and contrast-to-noise ratio.
• **in_vivo:** The *in vivo* directory contains all 20,121 acquisitions, which have been stored in compressed ZIP archives (one per volunteer). To maintain manageable file sizes, the data for Volunteer 011 is partitioned into five zip files. Each volunteer zip file contains multiple directories, one for each body area from which acquisitions were collected.

### 3.3 File format and structure

Each acquisition is saved as a compressed NumPy file (.npz). When loaded, each file contains the following arrays:

• **data**: Each acquisition is saved as a 3D NumPy array of raw RF data with shape [K x N_t_ x N_s_] = [105×192×2657]. The first dimension (K = 105) corresponds to the plane wave steering angles, the second dimension (N_t_ = 192) represents the physical receive transducer elements, and the third dimension (N_s_ = 2657) represents temporal samples.
• **body_region**: A string identifying the anatomical region (*e.g.*, “Abdomen”). In the case of the *in vitro* acquisitions, “full_speckle” for acquisitions taken on homogeneous speckle or “inclusions” for acquisitions taken on scatterers, hyperechoic or anechoic inclusions of the phantom.
• **volunteer_id**: An integer identifying the participant. The *in vitro* phantom has been assigned the volunteer ID 000.

### 3.4 Acquisition distributions and data characteristics

Table 2 summarizes the distribution of the 20,121 *in vivo* acquisitions across the six targeted anatomical regions. The volunteer-level data distribution across anatomical regions is detailed in Fig. 1, which provides the specific number of acquisitions collected per volunteer across each anatomical region. When using this dataset for machine learning studies, researchers are advised to adopt subject-wise evaluation protocols based on the volunteer identifiers to avoid potential data leakage.

**Table 2:** Summary of *in vivo* dataset distribution across anatomical regions.

Anatomical Region	Acquisition Count	Percentage (%)
Arms	4422	22.0%
Legs	4244	21.1%
Back	3246	16.1%
Breast	2874	14.3%
Neck (Carotid)	2736	13.6%
Abdomen	2599	12.9%
**Total**	**20,121**	**100%**

**Fig. 1:**
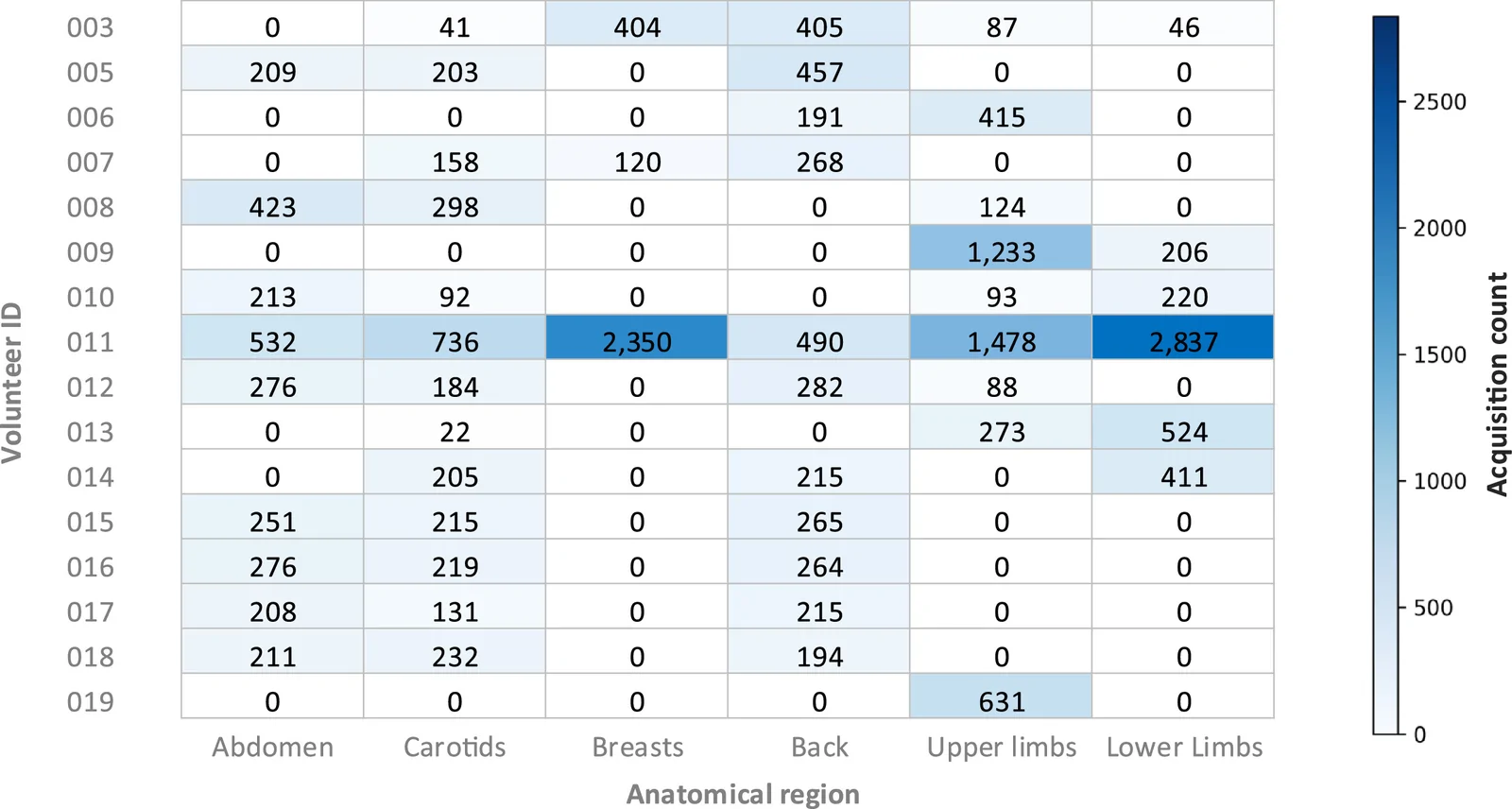
Volunteer-level acquisition distribution matrix across anatomical regions. The matrix summarizes the distribution of the 20,121 *in vivo* acquisitions across 16 volunteers and six anatomical body areas. Cell colors indicate acquisition density (darker blue represents higher volume).

To illustrate the data quality and variety, Fig. 2 provides representative B-mode images for each anatomical region and the CIRS 054GS phantom. These reference images were reconstructed using a standard DAS algorithm, implemented in the accompanying GPU-accelerated script (beamform.py). The figure is organized to show, in the first row, the images reconstructed using a single unsteered PW and, in the second row, images reconstructed using CPWC of all 105 steered angles, demonstrating the high-quality images achievable with this dataset.

**Fig. 2:**
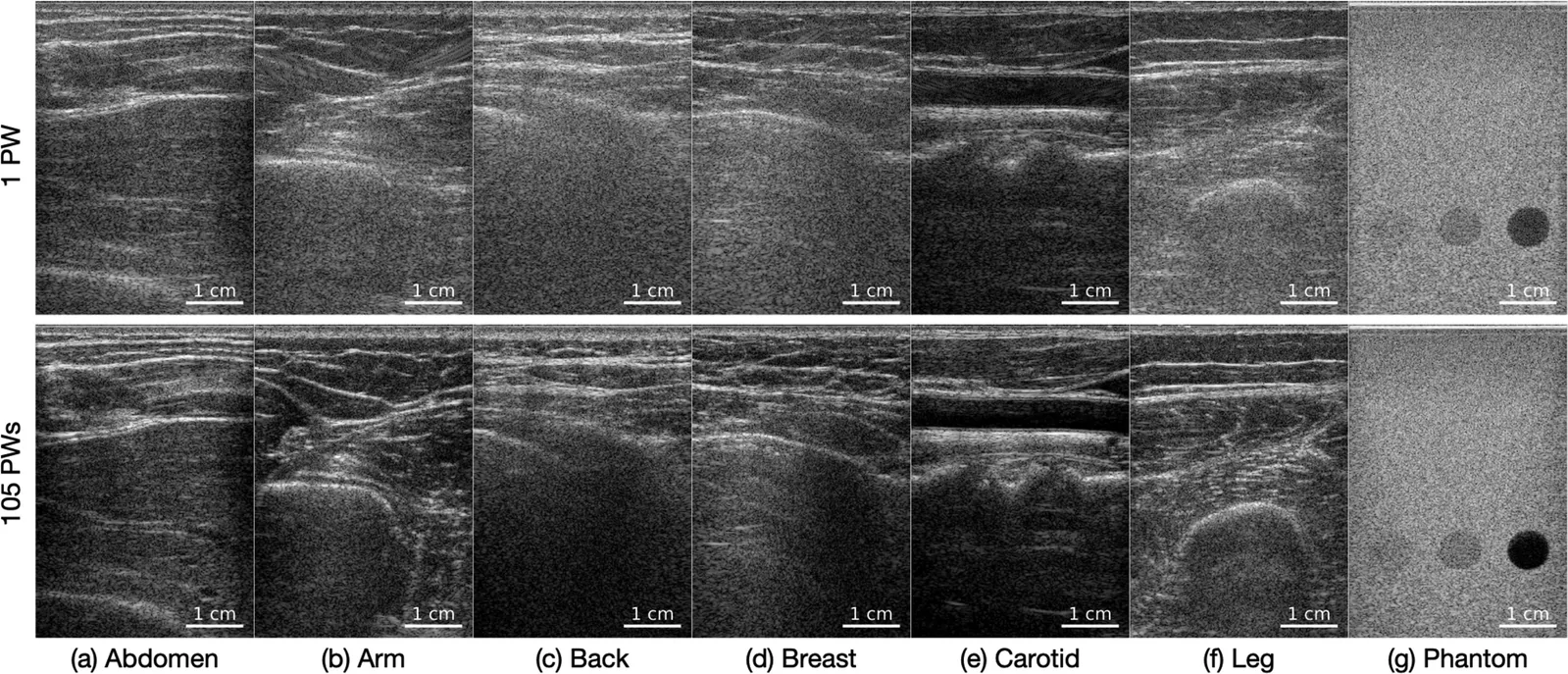
Representative B-mode images of the six anatomical regions and the CIRS 054GS phantom. Top row: Reconstructions from a single unsteered PW. Bottom row: High-quality images achieved via coherent PW compounding.

To ensure high diversity across the dataset and prevent frame redundancy, the ultrasound probe was manually repositioned to a unique spatial plane and distinct anatomical cross-section for every single acquisition. The dataset contains no continuous video streams or repetitive holds on identical planes.

The statistical distribution of the resulting raw RF signals is presented in Fig. 3. Subfigure 3(a) displays the probability density function of the raw RF amplitudes, while Subfigure 3(b) shows the distribution of the log-compressed magnitudes in decibels (dB), calculated as $20\;\text{lo}g_{10}\left(|RF_{value}|\right)$. Characterizing the global distribution and dynamic range of raw RF signals is essential for deep learning applications, as it directly informs pre-processing decisions such as data normalization, and loss function design [2,3].

**Fig. 3:**
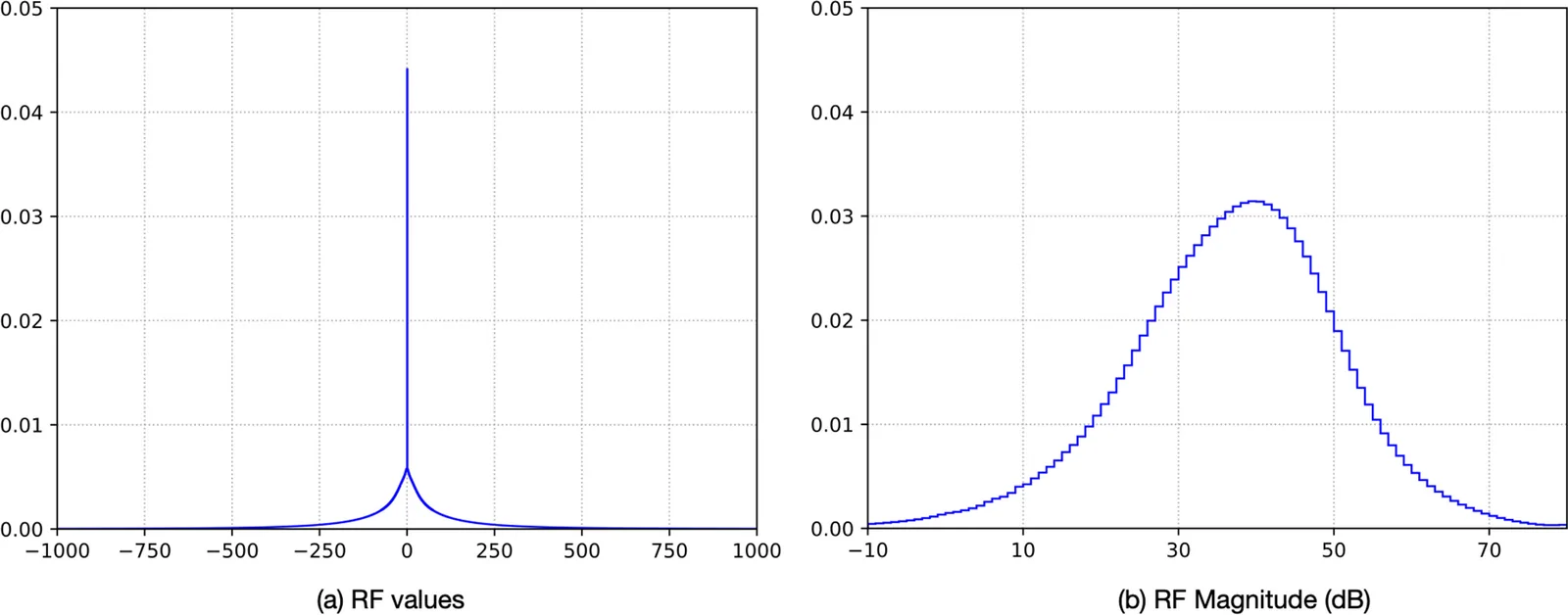
Probability density functions of the *in vivo* dataset. (a) Raw RF signal amplitudes across all 20,121 acquisitions. (b) Log-compressed signal magnitudes in decibels (dB).

## 4 Experimental Design, Materials and Methods

### 4.1 Participant characteristics and ethics

*In vivo* data were collected from 16 healthy volunteers (11 males, 5 females) aged 22–33 years. Acquisitions covered six anatomical regions as detailed in Table 2 and Fig. 1. All procedures were performed with the formal approval of the Cantonal Commission on Ethics in Human Research (Protocol 2022–01,696, CER-VD, Vaud, Switzerland). Written informed consent was obtained from all participants. Volunteer IDs (003–019) are synchronized with the dataset published in [2] to allow for comparative studies.

### 4.2 Acquisition hardware, sequence and safety

All data were acquired using a GE 11L-D linear array transducer connected to a Verasonics Vantage 256 system. The technical specifications of the probe include 192 transducer elements, a 200 µm pitch, a 180 µm element width, and an elevation focus at 17 mm. The system operated at a center frequency of 7.3 MHz with a 75% bandwidth.

A PW sequence was implemented using the Verasonics programming environment. Each acquisition sequence consisted of 105 PWs transmitted at a pulse repetition frequency of 9 kHz, using an alternating steering sequence with 0.32° increments. Transmissions were performed with a peak-to-peak voltage of 40 V, while digital time gain compensation (TGC) was applied to the received signals, assuming a standard soft-tissue attenuation coefficient of 0.5 dB/(cm·MHz) and a constant speed of sound of 1540 m/s.

Although 105 angles are provided, users wishing to replicate the study in [2] should utilize the 103 angles closest to 0° by discarding the two most extreme steering angles. This 103-PW configuration was selected to ensure focusing quality comparable to an optimal multi-focus setup with an F-number of 1.75 [2].

For *in vivo* data, the probe was repositioned to a new location after each acquisition. The programmed sequence imposed a maximum frame rate limit of 30 Hz across consecutive triggers, maintaining the Intensity Spatial Peak Temporal Average below the FDA-recommended threshold of 94 mW/cm². In practice, manual repositioning added significant time between acquisitions, making the actual interval much longer. A peak-to-peak voltage of 40 V was applied, resulting in a Mechanical Index (MI) below 0.7, compliant with BMUS guidelines.

### 4.3 Beamforming and image reconstruction

To facilitate data loading, image processing, and verification, a standalone Python module (beamform.py) is provided with the repository. This code generates B-mode images from the raw RF data implementing DAS and CPWC. The pipeline reads acquisition parameters directly from settings/beamforming_settings.yaml, computes transmit and receive propagation delays across the array elements on a 1/8λ spatial grid, and performs GPU-accelerated reconstruction. Analytic signals are generated via the Hilbert transform for envelope detection, followed by log-compression to a 60 dB dynamic range. Users can load raw RF tensors and reconstruct individual B-mode frames via command-line execution (*e.g.*, python beamform.py –acquisition path/to/acquisition_filename.npz).

## Limitations

While this dataset provides over 20,000 raw RF acquisitions across multiple body regions, several inherent limitations should be noted:

• Healthy Demographics & General Scope: Data were collected from a cohort of 16 healthy adult volunteers (aged 22–33). As a broad, general-purpose dataset, it is not tailored to clinical-specific tasks or pathological cases. However, it serves as a strong foundation for pre-training models that can subsequently be fine-tuned on smaller, clinical-specific datasets.
• Hardware Constraints: All data were acquired using a single linear array transducer (GE 11L-D) on one research platform (Verasonics Vantage 256). While single-hardware acquisition is an inherent constraint, releasing this collection complements our previously published GE 9L-D dataset [2], forming a paired public benchmark for researchers investigating cross-transducer domain adaptation and hardware-agnostic reconstruction for linear probes. Studies exploring non-linear geometries (*e.g.*, convex or phased arrays) must pair this dataset with external resources.
• Single Fixed Sequence: Although plane wave imaging is a standard benchmark sequence, all acquisitions were performed using a single fixed protocol (105 PWs). No focused wave acquisitions, other ultrasound sequences, or alternative steering angle configurations are included.
• Static 2D Frames: Each acquisition represents a single spatial cross-section rather than continuous, high-frame-rate temporal video sequences required for instance for dynamic motion tracking or functional imaging.

## Ethics Statement

The authors confirm that all research involving human subjects was carried out in accordance with the Declaration of Helsinki. Informed consent was obtained from all 16 participants prior to their inclusion in the study. All procedures and the experimental protocol were reviewed and formally approved by the Cantonal Commission on Ethics in Human Research (CER-VD, Vaud, Switzerland) under protocol number 2022–01,696.

## CRediT Author Statement

**Roser Viñals:** Conceptualization, Methodology, Software, Validation, Formal analysis, Investigation, Data Curation, Writing - Original Draft, Visualization, Funding acquisition; **Jean-Philippe Thiran:** Writing - Review & Editing, Supervision, Project administration, Funding acquisition.
